# Cadaveric Model of Pneumothorax (the GASP model): A Realistic, Reusable and Cost-Effective Simulator for Chest Drainage Training

**DOI:** 10.5070/M5.52367

**Published:** 2026-10-31

**Authors:** Thibault Planchamp, Audrey Soulet, Pierre Brouchet, Romain Vergé, Axel Rouch, Juliette Edme, Amaury de Barros, Mathieu Moré, Nathalie Galaup, Vanessa Danevoy, Fatima-Zohra Mokrane, Nicolas Guibert, Fanny Vardon, Élodie Chantalat, Thomas Geeraerts, Guillaume de Bonnecaze, Olivier Abbo, Charles-Henri Houzé-Cerfon, Patrick Chaynes, Luana Carfagna

**Affiliations:** 1 Toulouse University, Institute of Anatomy and Surgical Simulation (IASC), Toulouse, France; 2 Toulouse University Hospital, Hôpital des Enfants, Department of Pediatric Surgery, Toulouse, France; 3 Center of Anthropobiology and Genomics of Toulouse, Faculté de Médecine de Purpan, Toulouse, France; 4 Toulouse University Hospital, Hôpital Larrey, Department of Thoracic Surgery, Toulouse, France; 5 Toulouse University Hospital, Hôpital Rangueil, Department of Radiology, Toulouse, France; 6 Toulouse University Hospital, Department of Pulmonology, Toulouse, France; 7 Toulouse University Hospital, Hôpital Pierre-Paul Riquet, Department of Neurosurgery, Toulouse, France; 8 Toulouse University Hospital, Department of Anaesthesiology and Intensive Care, Toulouse, France; 9 Toulouse University Hospital, Hôpital Rangueil, Department of General and Gynaecological Surgery, Toulouse, France; 10 Toulouse University Hospital, Toulouse Institute for Health Simulation, Toulouse, France; 11 Toulouse University Hospital, Hôpital Larrey, Department of Oto-Rhino-Laryngology Head and Neck Surgery, Toulouse, France; 12 Toulouse University Hospital, Hôpital Pierre-Paul Riquet, Department of Emergency Medicine, Toulouse, France

## Abstract

**Audience:**

The gastrostomy-assisted simulated pneumothorax (GASP) model is designed to train residents in emergency medicine, anesthesiology, pulmonology, cardiothoracic surgery, and paediatric surgery in the various techniques used to perform chest drainage (CD).

**Introduction:**

Pneumothorax is a life-threatening emergency, particularly in the case of a tension pneumothorax. Failure to perform timely CD can rapidly result in cardiorespiratory arrest.^1^ Needle decompression, chest tube insertion, and finger thoracostomy are the three main CD procedures that healthcare professionals must master. However, these are technically challenging procedures with potentially severe and sometimes fatal complications. The literature reports significant complication rates (19%) and failure rates (18–21%).^2^,^3^

Synthetic simulators for CD are commonly used in simulation centres due to their availability. While they are useful in the early stages of training to teach the fundamental steps of the procedure, they lack realism, are expensive, and do not provide the tactile feedback essential for safe and effective performance. The GASP model was developed to provide a realistic, easily reproducible, and accessible pneumothorax simulation tool aimed at improving CD training for medical trainees.

**Educational Objectives:**

By the end of this educational session using the GASP model, learners should be able to: 1) identify the two main thoracic anatomical landmarks commonly used in clinical practice for CD: the second intercostal space at the midclavicular line, and the fifth intercostal space at the midaxillary line, 2) become familiar with the tactile sensation of palpating the superior border of the rib, a key landmark for directing the chest tube safely and with the correct angulation, 3) mastering the three main techniques of CD: needle decompression, chest tube insertion, and finger thoracostomy, and 4) demonstrate proficiency while performing the procedure within a complex simulation scenario.

**Educational Methods:**

The GASP model utilises fresh cadavers prepared with an insufflation device and a gastrostomy button to simulate pneumothorax or haemopneumothorax. Manual or automated ventilation may be added to enhance realism. This model can be used to teach residents how to perform the three main CD procedures effectively and safely. Each learner can perform multiple repetitions on each cadaver.

**Research Methods:**

At the Institute of Anatomy and Surgical Simulation (IASC) in Toulouse, France, 10 fresh cadavers preserved by refrigeration were used for this study. Each donor had provided informed and written consent during their lifetime for the use of their body in experimental research. Demographic data related to the cadavers, such as age, sex, and body mass index, were collected.

The GASP model was tested on 20 hemithoraces from the 10 cadavers. The presence and quality of the pneumothorax were assessed via ultrasound by an experienced radiologist. The two main CD approaches commonly used in clinical practice (axillary and anterior) were tested, along with several CD techniques: needle decompression, direct insertion using a Pleurocath^®^ drain and Monod trocar, the Seldinger technique with a Fuhrman-Cook drain, and finger thoracostomy.

The GASP model was subsequently introduced to first-year residents in anesthesiology, emergency medicine, and pulmonology. Participants used Likert scales to evaluate the model’s realism, its transferability to clinical practice, and its overall educational value. Additionally, the study assessed learners’ perceived self-efficacy and the level of discomfort associated with performing simulations on cadaveric specimens.

**Results:**

In all 20 hemithoraces (10 right and 10 left), a large pneumothorax was confirmed by ultrasound. All major CD techniques were successfully performed, using a variety of drainage devices appropriate to different clinical scenarios. The model provided a realistic simulation of the procedure, offering the same tactile sensation as on a live patient. It enabled repeated CD in each hemithorax (exceeding 40 procedures per side) without compromising the structural integrity of the model.

Eight first-year residents (from anesthesiology, emergency medicine, and pulmonology) were enrolled in the first CD course on cadaver-based training at our faculty, using the GASP model. Pre- and post-training assessments based on 10-point questionnaires demonstrated significant improvements in baseline confidence in technical skills, stress management, and overall performance. The GASP model was also evaluated by participants using a 5-point Likert scale, revealing high levels of realism, strong transferability to patient care, and increased perceived self-efficacy among learners. Finally, the cadaveric model was associated with minimal discomfort for participants.

**Discussion:**

The GASP model is realistic, easy to implement, reproducible, low-cost, and reusable multiple times. It serves as a valuable tool for enhancing skill acquisition among a wide range of healthcare professionals who need to master this procedure, providing a level of tactile realism that is often lacking in synthetic models. Its successful integration within our medical simulation centre supports the development of additional cadaveric simulation models for other emergency procedures.

**Topics:**

Pneumothorax, chest drainage, emergency medicine, simulation, anatomy.

## USER GUIDE

**Table:** 

List of Resources:	
Abstract	18
User Guide	21
Appendix A: PowerPoint Presentation	26

**Learner Audience:**

Medical students, interns, and residents in emergency medicine, anesthesiology, pulmonology, thoracic surgery, and paediatric surgery.

**Time Required for Implementation:**

- **Construction of the GASP model**: 4 to 7 minutes per hemithorax were required to set up the model, excluding the insufflation step. No advanced surgical skills were required.
- **Learner time per drainage procedure**: approximately 5 minutes per learner.
- **Total time required for a learner to achieve procedural competence**: A half-day session with multiple workshops (eg, one for needle decompression, one for chest tube insertion, etc.) appears sufficient to achieve procedural competence and to conduct a debriefing of each procedure with the instructor.

**Recommended Number of Learners per Instructor:**

One instructor per 6 learners. Two learners can train simultaneously on the same cadaver (1 on each hemithorax).

**Topics:**

Pneumothorax, chest drainage, emergency medicine, simulation, anatomy.

**Objectives:**

By the end of this educational session, using the GASP model, learners should be able to:

1. Identify the two main thoracic anatomical landmarks commonly used in clinical practice for CD: the second intercostal space at the midclavicular line, and the fifth intercostal space at the midaxillary line,^4^
2. Become familiar with the tactile sensation of palpating the superior border of the rib, a key landmark for directing the chest tube safely and with the correct angulation,^4^
3. Mastering the three main techniques of CD: needle decompression, chest tube insertion and finger thoracostomy,^5^
4. Demonstrate proficiency while performing the procedure within a complex simulation scenario.^6^

### Linked objectives, methods and results

The pedagogical framework and instructional methods were developed to support the acquisition of both technical and non-technical competencies required for proficient CD management. Through a combination of expert-led theoretical reviews, explicit skills objectives (PowerPoint), and high-fidelity cadaveric simulation, participants systematically enhance their anatomical understanding, procedural confidence, and mastery of CD techniques.

The use of the GASP model provides highly realistic anatomical and tactile feedback, allowing repetitive deliberate practice in conditions closely reproducing real-life clinical situations. Rotating through several dedicated stations also promotes active participation and immediate application of knowledge acquired during the introductory teaching sessions. The conceptual framework underlying this educational intervention is based on Kolb’s Experiential Learning Theory and constructivist learning principles, emphasizing experiential learning, active experimentation, and reflective debriefing to enhance skills retention and transferability to clinical practice.

### Recommended pre-reading for instructor

Instructors are encouraged to review thoracic anatomy and procedural techniques using authoritative resources to facilitate the preparation of the GASP model and to effectively teach the objectives, including the risks of serious complications involving neighboring anatomical structures. Recommended resources include the *Netter Atlas of Human Anatomy* and the *Roberts and Hedges’ Clinical Procedures in Emergency Medicine and Acute Care.*^*8,9*^ Furthermore, the video provided with this article (Video 1: Step-by-step guide to building and using the GASP model) serves as a comprehensive guide for the instructor throughout the creation of the GASP model.

### Learner responsible content (LRC)

The simulation session was preceded by 10-minute didactic presentations delivered by four senior physicians from various specialties at our institution (an anatomist, a pulmonologist, an anesthesiologist, and a thoracic surgeon). These voluntary lectures covered the following topics: thoracic anatomy, the pathophysiology of pneumothorax, indications and contraindications for chest tube placement, and an overview of currently available drainage techniques and devices.

Should specialist physicians be unavailable to provide these theoretical refreshers, learners are expected to independently review the following topics and resources prior to the simulation to ensure optimal workshop conditions:

- Thoracic anatomy (eg, *Netter Atlas of Human Anatomy*);^8^
- The pathophysiology of pneumothorax;^10^
- Current guidelines regarding pneumothorax management strategies and associated equipment.^9^,^11^,^12^

### Implementation Methods

The instructional session using the GASP model is conducted over a half-day period at the Institute of Anatomy and Surgical Simulation (IASC) in Toulouse, France.

Initially, learners are briefed on the four specific learning objectives they are expected to master by the end of the program. The introduction also covers the ethical considerations regarding cadaveric use, hygiene protocols, and the technical specifications of the GASP model.

Then, a review of the fundamental knowledge required to perform CD procedures through four teaching modules are made: thoracic anatomy (this presentation may be supported by cadaveric dissection or slides to enhance clarity and engagement), the pathophysiology of pneumothorax, indications and contraindications for CD, and an overview of currently available drainage techniques and devices. These theoretical refreshers may be delivered as didactic lectures by senior physicians at the beginning of the training program. Should specialist physicians be unavailable to provide these sessions, learners are expected to independently review these four topics prior to the simulation day using the Learner Responsible Content (LRC) materials.

Finally, participants rotate through 4 structured hands-on stations:

- a GASP model for needle decompression
- a GASP model for chest drain insertion using either the Seldinger technique or a direct surgical approach (e.g. Pleurocath^®^)
- a GASP model for finger thoracostomy with manual exploration of the pleural cavity, including clot evacuation in the context of a haemopneumothorax
- and a GASP model with high-fidelity emergency simulation using the SIMLIFE^®^ system, in which participants manage a tension pneumothorax or haemopneumothorax on a re-ventilated and recirculated cadaver.^6^

Each station enables trainees to practise technical skills directly on the cadaveric model, which offers highly realistic tactile and anatomical feedback. The model can be reused multiple times after re-insufflation of the hemithorax. All procedures are carried out under close supervision by experienced instructors.

The session concludes with a structured debriefing designed to consolidate learning outcomes, address remaining questions, and introduce a training portfolio. Trainees are encouraged to document the outcomes, both successful and unsuccessful, of subsequent CD procedures performed in clinical practice. This logbook will serve as the foundation for prospective studies to evaluate whether training with the GASP model effectively reduces procedural failure and complication rates among residents.

### List of items required to replicate this innovation

1. **Mic-Key**^**®**^ **Enfit Low Profile Gastrostomy Feeding Tube Kit**: Approximately $150 per unit for a brand-new kit (source: medicaleshop.com). However, this cost can be avoided by utilizing expired kits obtained in collaboration with the hospital’s stoma therapy department.
2. **Sims Tubing Connector Argyle**^®^ **3/16″– 7/16.″** Estimated cost of $1.82 per unit (source: medicaleshop.com).
3. **UROMED**^®^ **nephrostomy adapter 2675 Luer-Lock to funnel** (estimated cost of $15 per unit on ebay.com) or **Porgés**^®^ **connecteur Luer embout entonnoir cat.urét** (source: publicare.ch).
4. **Three-way stopcock Luer lock adapter**. Estimated cost of $0.63 per unit (source: AliExpress).
5. **Optional Tubing Extension:** If additional length is required, a segment of plastic tubing can be added between the three-way stopcock and the Luer Lock syringe.
6. **60 mL Luer Lock Syringe:** Estimated cost of $1.00 per unit (source: Amazon).
7. **Surgical Blade:** Estimated cost of $0.18 per unit (source: Amazon).
8. **2/0 Suture Thread:** Estimated cost of $0.99 per unit (source: AliExpress).

### Approximate cost of items to create this innovation

Excluding cadaver-related costs inherent to the operation of an anatomy laboratory, each new Mic-Key^®^ Enfit Low Profile Gastrostomy Feeding Tube Kit costs approximately $150. However, the use of a brand-new kit is not necessary. By collaborating with the hospital’s stoma therapy department to obtain expired gastrostomy kits, this innovation can be implemented at no additional cost.

### Detailed methods to construct this innovation

1. Make a 1.5 cm incision in the 4th intercostal space, on either the right or left side, at the level of the midclavicular line. Extend the incision down to the endothoracic fascia (Figure 1).
  **Figure 1:**
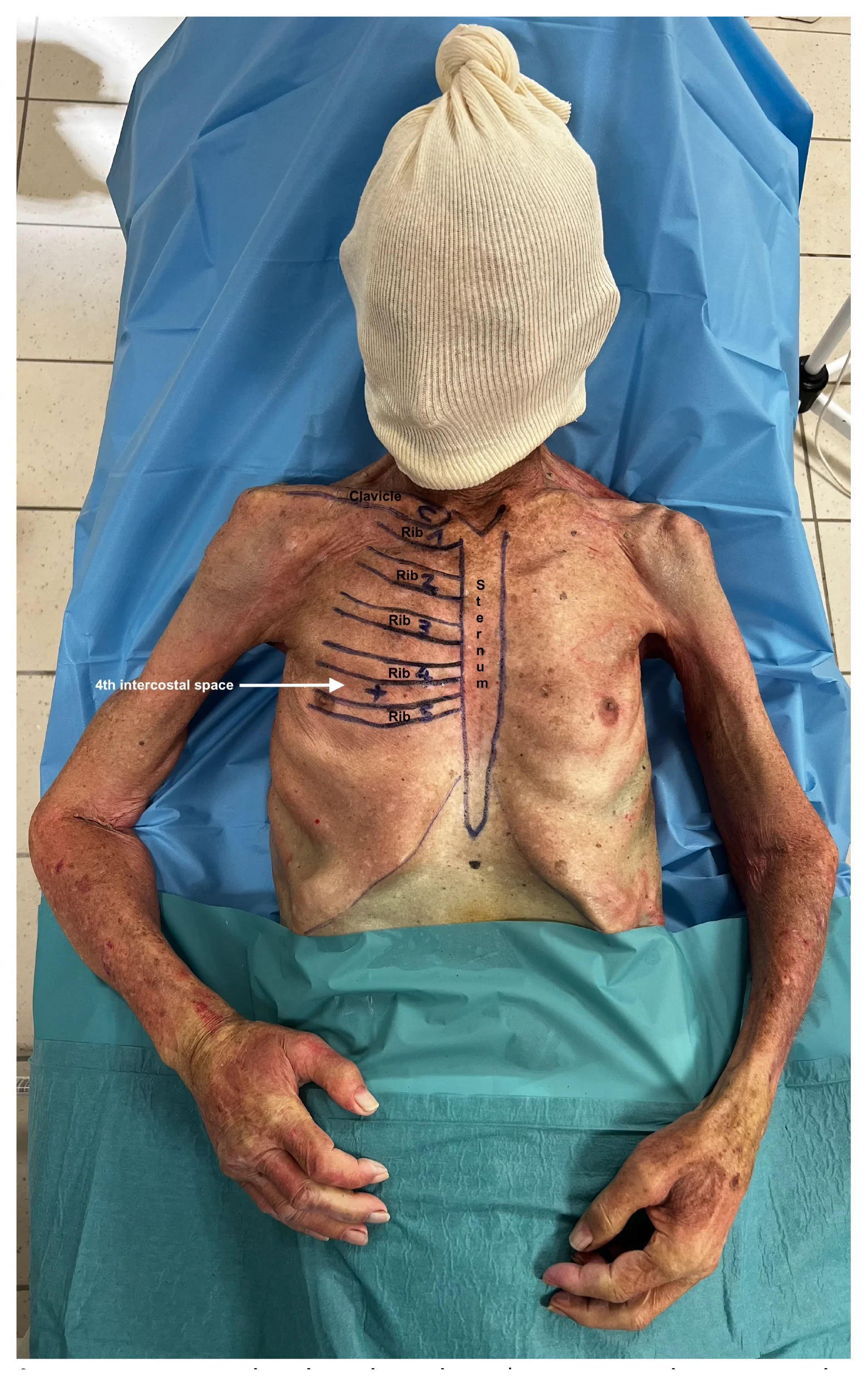
Incision landmark in the 4^th^ intercostal space at the midclavicular line. The clavicle, sternum, and ribs were also identified. Author’s own image.
2. Perforate the endothoracic fascia and the underlying parietal pleura using the index finger. To facilitate pleural detachment during insufflation, sweep the index finger in a 360° rotational motion around the opening to break any pleural adhesions.
3. Insert the gastrostomy balloon through the intercostal incision and advance it into the pleural cavity.
4. Place a purse-string suture using 2/0 suture thread around the cutaneous opening of the gastrostomy to ensure an airtight seal of the balloon.
5. Inflate the gastrostomy balloon with 10 mL of air.
6. Connect the gastrostomy button to its dedicated adaptor, trimming the distal tip to allow attachment to the Sims Tubing Connector Argyle^®^ This connector is then linked to a three-way stopcock via the UROMED^®^ nephrostomy adapter or the Porgés^®^ Luer connector. If additional length is required, a segment of plastic tubing can be added between the three-way stopcock and the Luer Lock syringe. Complete assembly is illustrated in Figure 2 and detailed step-by-step in Video 1.
  **Figure 2:**
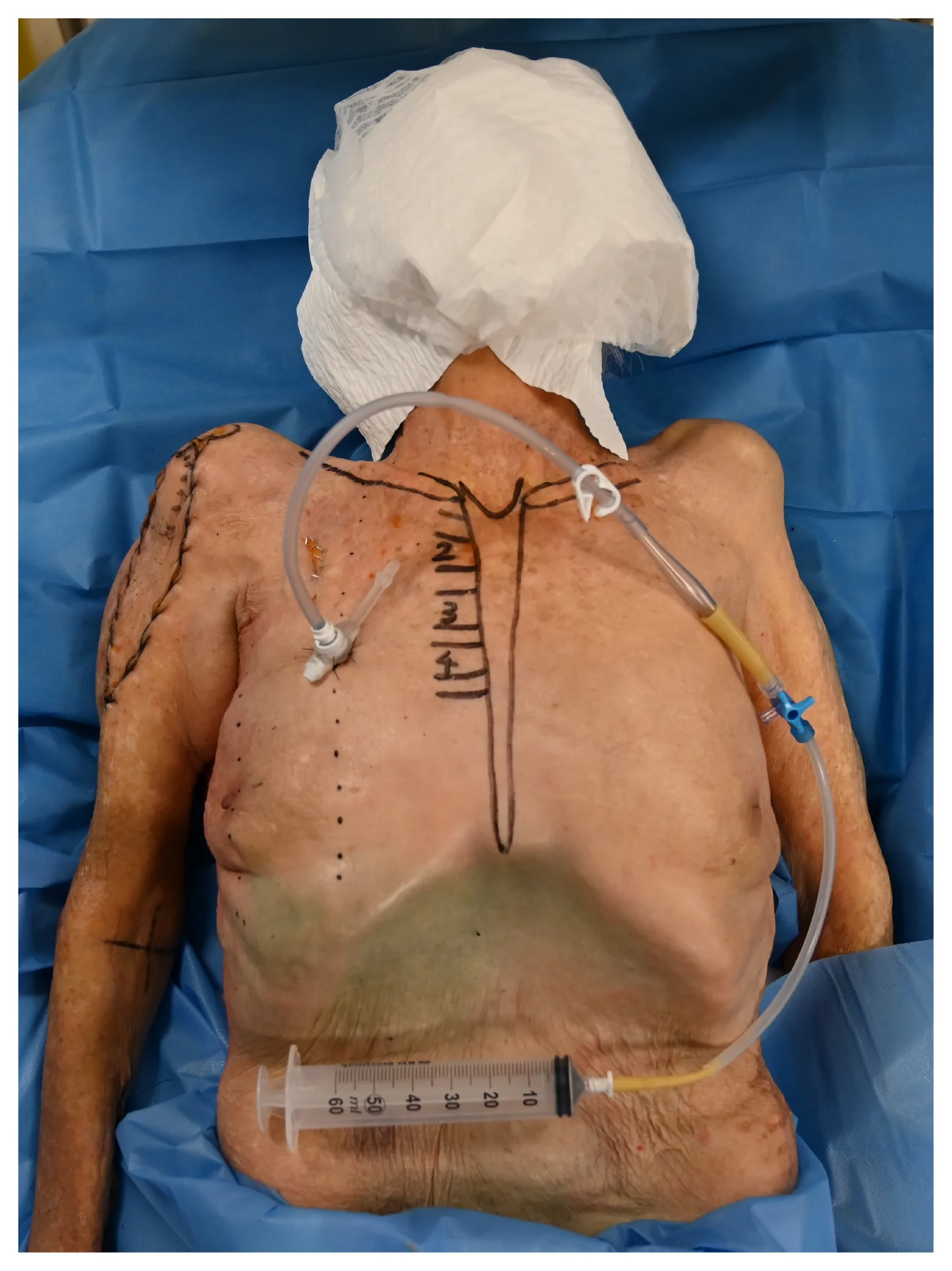
Completed setup of the GASP model. Author’s own image. **Video 1.** Step-by-step guide to building and using the GASP model. https://youtu.be/VIuKkJeTLhA
7. Using a Luer Lock syringe, insufflate 1 liter of air into the hemithorax to simulate a pneumothorax. The three-way stopcock allows both air insufflation and syringe refilling without detaching it from the assembly. The quality of the pneumothorax can be assessed via ultrasound. To simulate haemothorax drainage, fill the syringe with artificial blood instead of air.
8. Optionally, to enhance the realism of the model during drainage, a tracheostomy cannula may be surgically placed approximately 2 cm above the jugular notch. The cannula can then be connected either to a bag-valve mask for manual ventilation or to the SIMLIFE^®^ system for automated ventilation.

### Results and tips for successful implementation

#### Part 1: General Data

Ten fresh cadavers were used, allowing the creation of our pneumothorax model on 20 hemithoraces (10 right and 10 left). The sample consisted of 5 women and 5 men, with median age, weight, height, and BMI values of 85.5 years [range: 56–100], 64.5 kg [range: 57–95], 165 cm [range: 150–183] and 22.5 kg/m^2^ [range: 20–34.9] respectively.

The median duration for setting up the insufflation device (excluding insufflation itself) was 4.3 minutes [range: 3.5–7], requiring no advanced surgical skills.

#### Part 2: Quality of the Pneumothorax Model

At the end of the insufflation process, an air test confirmed the absence of leaks at the suture site. In case of leakage, an additional purse-string suture can be placed around the cutaneous opening of the gastrostomy to improve the airtightness of the model.

The quality of the pneumothorax was assessed via ultrasound by an experienced radiologist using the Mindray^®^ TE7 2019 ultrasound system (Mindray Bio-Medical Electronics Co.) (Figure 3).^7^ In all eight hemithoraces, a large pneumothorax was observed with pleural detachment along the entire height of the axillary line, indicating the need for thoracic drainage. In cases where satisfactory pleural detachment is difficult to achieve due to extensive pleural adhesions, this detachment can be improved by inserting a long, foam-tipped instrument into the pleural space and performing rotational movements within the space to break these adhesions and separate the lung from the chest wall.

#### Part 3: Thoracic Drainage Procedure

The two main CD approaches commonly used in clinical practice (the 2^nd^ intercostal space along the midclavicular line and the 5^th^ intercostal space along the midaxillary line) were tested. The model allowed both approaches to be performed without difficulty. The following CD methods were also evaluated: needle decompression, direct insertion using a Pleurocath^®^ drain and Monod trocar, the Seldinger technique with a Fuhrman-Cook drain, and finger thoracostomy. The model successfully facilitated CD procedures for each of these techniques. For example, in an axillary CD procedure using a Pleurocath^®^ drain, local anesthesia with Xylocaine was administered, followed by a 1 cm skin incision with a surgical blade in the 5^th^ intercostal space along the midaxillary line. The Pleurocath^®^ drain was then inserted toward the contralateral shoulder without difficulty. The drain was advanced, and the guidewire was removed. The evacuation of air was confirmed by an air test. A post-drainage ultrasound confirmed the successful evacuation of air (Video 1). After drain removal, the hemithorax was re-insufflated with 1 liter of air, and no major air leaks were detected. Only minor subcutaneous emphysema was observed at the drainage site, a common finding in clinical practice, especially in cases of traumatic pneumothorax associated with rib fractures. Chest drainage and re-insufflation procedures were successfully repeated over 40 times without compromising the model’s integrity. For other CD techniques, similar observations were made, except for the finger thoracostomy method, where an X-shaped skin suture was required at the end of the procedure to restore hemithorax airtightness and allow further insufflations.

#### Part 4: Results from users

The initial GASP model session included eight first-year residents (three anesthesiology, three emergency medicine, two pulmonology) with a median age of 24.5 years [range: 23–30] (62.5% male). Notably, none had prior formal training in CD.

Based on a 10-point pre-training questionnaire, participants reported low baseline confidence in technical skills (median 3, interquartile range (IQR) 2–4.3), stress management (median 5, IQR 4.5–5.3), and overall performance (median 3, IQR 2–5), alongside low apprehension toward using cadaveric models (median 3.5, IQR 1.5–6.3).

Post-training evaluation demonstrated significant improvements in self-assessed technical skills (median 8, IQR 7.8–9; *P* = 0.0002), stress management (median 8, IQR 8–9; *P* = 0.0002), and overall performance (median 8, IQR 8–9; *P* = 0.0002). Using a 5-point Likert scale, participants rated the GASP model as highly realistic (median 4.5, IQR 4–5), highly transferable to patient care (median 5, IQR 4–5), supportive of learners’ perceived self-efficacy (median 4, IQR 4–5), and pedagogically valuable (median 5, IQR 4–5). Discomfort related to the cadaveric model remained low (median 1.5, IQR 0–3.5, out of 10).

### Associated content

- Appendix A: PowerPoint Presentation
- Video: https://youtu.be/VIuKkJeTLhA

**Figure 3:**
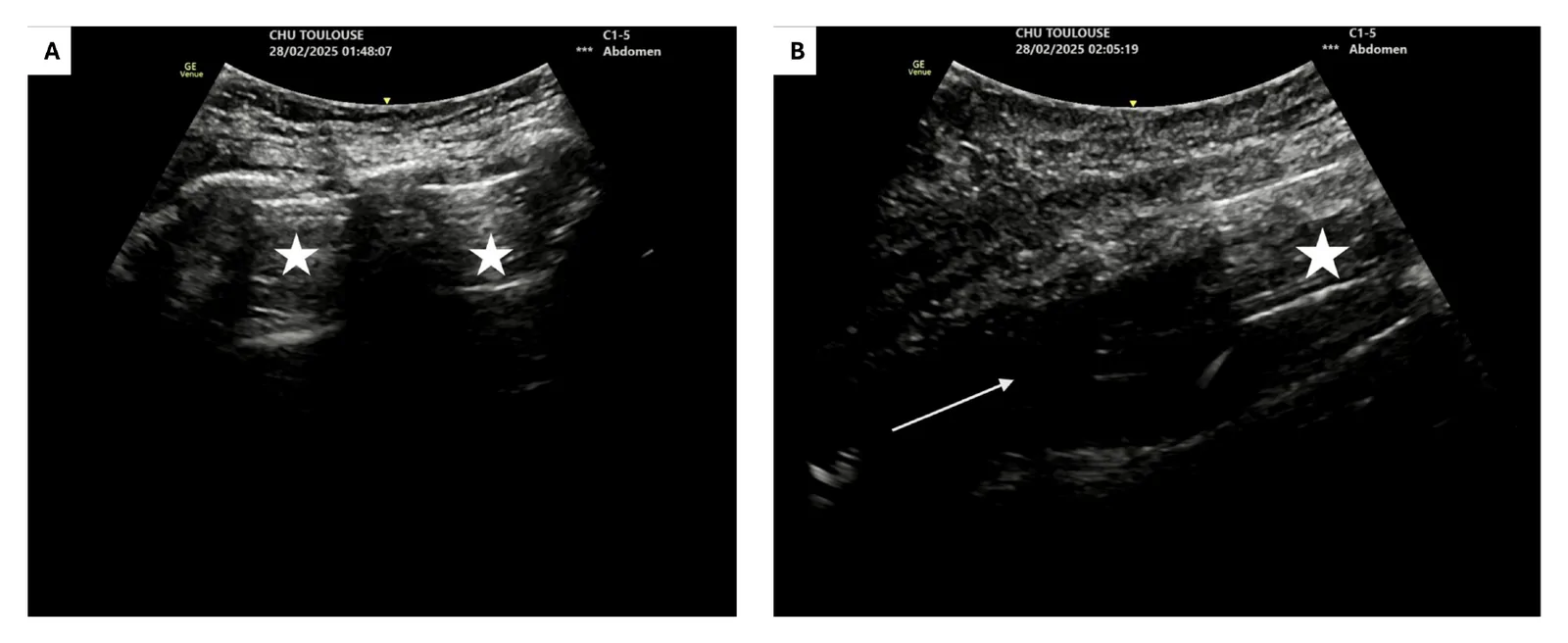
**A.** Ultrasound section of the anterior chest wall in a sagittal axis: aerial artifacts of a pneumothorax (stars). **B.** Ultrasound section through an intercostal space: collapsed and sloping lung parenchyma (arrow), aeriform artifact of pneumothorax (star). Author’s own image.

## Supplementary Information


